# Reflections on Resilience during the COVID-19 Pandemic: Six Lessons from Working in Resource-Denied Settings

**DOI:** 10.4269/ajtmh.20-0274

**Published:** 2020-04-17

**Authors:** Leah Ratner, Rachel Martin-Blais, Clare Warrell, Nirmala P. Narla

**Affiliations:** 1 Division of Pulmonary Medicine, Boston Children’s Hospital, Harvard Medical School, Boston, Massachusetts;; 2 Division of General Internal Medicine, Brigham and Women’s Hospital, Boston, Massachusetts;; 3 Division of Pediatric Infectious Diseases, University of California Los Angeles, Los Angeles, California;; 4 Department of Infectious Diseases, Royal Free NHS Hospitals Trust, London, United Kingdom;; 5 Division of Medical Critical Care, Boston Children’s Hospital, Harvard Medical School, Boston, Massachusetts

## Abstract

The 2019 novel coronavirus disease (COVID-19) pandemic highlights the experience of communities in the global South that have grappled with vulnerability and scarcity for decades. In the global North, many frontline workers are now being similarly forced to provide and ration care in unprecedented ways, with minimal guidance. We outline six reflections gained as Western practitioners working in resource-denied settings which inform our current experience with COVID-19. The reflections include the following: managing trauma, remaining flexible in dynamic situations, and embracing discomfort to think bigger about context-specific solutions to collectively build back our systems. Through this contextualized reflection on resilience, we hope to motivate strength and solidarity for providers, patients, and health systems, while proposing critical questions for our response moving forward.

The 2019–2020 novel coronavirus disease (COVID-19) pandemic, caused by the worldwide spread of severe acute respiratory syndrome coronavirus 2 (SARS-CoV-2), has changed healthcare systems across the globe. Many providers in high-income countries who have hardly known shortage in their lifetimes are being forced to consider triage, rationing, and altered provision of care in unprecedented ways. In the global North, this development has introduced frontline workers to vulnerability at a national level for the first time. Critical supply shortages of personal protective equipment (PPE) and ventilators have left healthcare workers (HCWs) with feelings of anxiety, guilt, and helplessness. However, these health system gaps may also highlight a source of newfound empowerment for change, as hardship can bring communities together to mobilize resilience. How can we, as HCWs from the global North, use this experience to learn new forms of resilience during this global health challenge? In the following text, we discuss collective lessons from global health work in resource-limited settings that inform our current experience with COVID-19.

## LESSON 1. WHAT FLEXIBILITY REALLY MEANS

Global health practitioners are taught that when working in a resource-constrained setting, effective approaches need to use locally driven solutions, informed by evidence that is context-specific. Although it is tempting to apply previous experiences to new situations, context may make previous solutions irrelevant. For example, it may not be ethical to perform cardiopulmonary resuscitation in settings where ventilators and circulatory supports are not available to support the patient in the post-resuscitation period. Identifying resource-specific solutions focused on equity, efficiency, and sustainability of the health system thus becomes essential in.

In a resource-limited setting, flexibility means adapting your practice in dynamically changing situations. This often involves pushing yourself outside of your comfort zone to focus on interpersonal and systematic details rather than stylistic ones. True flexibility can be astoundingly hard to achieve, and requires a fund of humility that we often lack in the medical community. However, a willingness to approach challenges in new ways—rather than trying to fit them to a previous mold—allows for solutions to materialize. We reflect on the increasing importance to recognize (and amplify) locally adapted successes when working in unfamiliar settings, acknowledging that this level of change is often uncomfortable.

## LESSON 2. FIND EXPERTISE IN PLACES YOU MAY NOT HAVE THOUGHT TO LOOK BEFORE

In the global North, we are accustomed to accessing expert guidelines that dictate how we practice medicine. However, this routine reliance to inform best practice has been dismantled in the setting of COVID-19 because in a novel situation, no one knows with certainty how best to proceed—at every level of every organization. Our inability to perform this kind of informed decision-making contributes to new feelings of anxiety, both within the medical community and amongst the lay public.

In this time of uncertainty, we have found ourselves drawing on lessons learned from our colleagues in the global South: creative ways to reuse and make PPE, new methods for sterilizing limited resources, and new treatment modalities that bypass high-cost medications. When clinically applicable, minimize hierarchy to facilitate engagement of all stakeholders—patients, HCWs, and community members—in bidirectional learning and creative problem-solving. Many of the most ingenious solutions to difficult problems come from colleagues who regularly think outside the box to overcome resource limitations and drive context-specific solutions. As the line between individual sub-specialties begins to blur and hierarchy is flattened—as chiefs of surgery are now being asked to practice as internal medicine interns on COVID-19 wards—new experts are quickly emerging. Let us look forward to calling on the newly gained specialty-agnostic expertise of COVID-19 providers in New York City, London, Wuhan, Milan, and Madrid.

## LESSON 3: BLEND INTERPERSONAL RELATIONSHIPS AND WORK; ADAPT YOUR PERCEPTION OF TIME

In Western cultures, monochronic time, or the sense that time is a commodity—limited and thus valuable—is a commonly shared ideal. Although monochronic time breeds effective multitasking by minimizing “waste,” it also places relative values on each of our relationships (time spent being proportional to importance in our lives). When demands on time are high, the ability to manage ever-increasing patient loads and academic requirements is viewed as a productive skill; however, this approach limits our engagement in any one area.

Variation in this conception of time can cause stress for first-time global health practitioners. Many cultures in the global South are polychronic,^2^ where time is viewed as infinite. Like a stream, polychronic time adjusts its shape to fill the available space. It allows for meetings to seamlessly flow in and out of personal connection, and is often embraced in cultures where uncertainty is commonplace, allowing the *unexpected to be expected*. Deadlines are fluid, and empathy for understanding how life’s struggles influence these deadlines is recognized collectively. This understanding frees individuals from the tyranny of time, allowing focus where it is most needed in the moment. Although one’s conception of time is difficult to alter, acknowledging the fluidity of time may help us adapt in a rapidly changing environment.

## LESSON 4. USE CHALLENGING SITUATIONS AS OPPORTUNITIES TO ADVOCATE

Like many disasters before it, the COVID-19 pandemic is bringing to light long-standing structural oppression, embedded racism, and widespread class inequity. Recent CDC data show that black Americans were more likely to require hospitalization for COVID-19 than white Americans in the same catchment area, and early data suggest they may also have higher rates of death.^3,4^ Although freedom of social mobility caused resource-replete countries to be the first to be affected by the novel virus’s spread, they will hardly have the worst experience if communities with fewer resources are left to deal with the fallout on their own.

As social medicine advocates, we have consistently seen that those who are most vulnerable require increased, not equal, support in times of crisis to achieve equity. It is imperative to openly discuss the magnitude of disparity this pandemic is exposing, rather than diminish it. Epidemiologists and public health specialists, policy advocates, scientists, and frontline HCWs—no matter what your role, now is your time to advocate for solutions to injustice, poor safety planning, and inequity. This advocacy feels particularly uncomfortable during uncertain times, but remember that uncertainty exists because the system is broken. Recognize your strengths and where you can contribute in the call for progress.

## LESSON 5. BEING A HCW WHEN RESOURCES ARE SCARCE IS TRAUMATIC

“Trauma-Informed Care”^1,5^ has never been more critical. The existing literature largely focuses on coping strategies for HCWs outside of work, but what about during work, in the face of extraordinary demands? What about when friends and family call during “off hours” to ask a litany of questions about severe acute respiratory syndrome coronavirus 2 and its implications?

We tell our first-time global health practitioners to practice *radical vulnerability*^6^ by allowing themselves to be openly honest about their needs with family, friends, and clinical colleagues. We are reminded to think about what our strengths and weaknesses look like at the peak of stress and adjust with individually tailored coping mechanisms, curated over years of training. Novel coronavirus disease feels no different, but this time, we were stripped from the ability to pre-prepare. Now, it is more important than ever to recognize your own trauma and to tell your coworkers when you are nearing burnout, not sleeping, or emotionally exhausted. In this era, assume that everyone comes from a place of trauma; listen from that place and share in that same vulnerability. Teach yourself to optimize resilience by preserving your boundaries, and ask your friends and family to respect your “virus-free time.”

## LESSON 6. DO LOOK BACK–THE RIGHT WAY

No one denies that the COVID-19 pandemic has placed us in an unprecedented and terrifying situation. Yet how can we move beyond the frustration at our lack of preparedness and toward channeling our energy productively despite resource limitations?

Many in this situation may attempt to take on the fragility of the health system single-handedly, only to be quickly overwhelmed.^7^ Despite the prominent role of the Plan-Do-Study-Act cycles in quality improvement literature,^8^ many healthcare systems do not allow sufficient time for personal self-reflection. Yet a resilient healthcare provider finds time to reflect on past mistakes, grow as a practitioner, and learn to avoid future pitfalls. Without learning from individual and systemic failures, we would never improve. Remind yourself and your colleagues daily that you are one piece in a giant global community working to turn the tide. Most of this cannot be controlled by you, so concentrate on where you can effect change. Do not live in the past, but learn from your experiences moving forward.

## CONCLUSION

As Western-trained clinicians, how will working and living through the COVID-19 pandemic change our role in health care? How will it impact our view of the global community and give us a new empathic understanding for scarcity? How will recognition of our own embedded trauma allow us to better take care of others? How will we work to dismantle oppression in our health system at a regional, national, and international level? How can we work together to ensure preemptive health system strengthening and effective rebuilding? Although it may have taken a pandemic to publicly expose the vast interconnectedness of our global community, only by continuing to learn from this interdependence can we build back our collective society stronger.
